# iPSC-based approach for human hair follicle regeneration

**DOI:** 10.3389/fcell.2023.1149050

**Published:** 2023-05-30

**Authors:** Chinnavuth Vatanashevanopakorn, Thanutchaporn Sartyoungkul

**Affiliations:** ^1^ Department of Biochemistry, Faculty of Medicine Siriraj Hospital, Mahidol University, Bangkok, Thailand; ^2^ Siriraj Center for Regenerative Medicine, Faculty of Medicine Siriraj Hospital, Mahidol University, Bangkok, Thailand

**Keywords:** hair follicle regeneration, induced pluripotent stem cells, dermal papilla cells, hair follicle stem cells, skin organoids

## Abstract

Hair follicles (HFs) are a multifunctional structure involved in physical protection, thermoregulation, sensational detection, and wound healing. Formation and cycling of HFs require dynamic interaction between different cell types of the follicles. Although the processes have been well studied, the generation of human functional HFs with a normal cycling pattern for clinical utilization has yet to be achieved. Recently, human pluripotent stem cells (hPSCs) serve as an unlimited cell source for generating various types of cells including cells of the HFs. In this review, HF morphogenesis and cycling, different cell sources used for HF regeneration, and potential strategies for HF bioengineering using induced pluripotent stem cells (iPSCs) are depicted. Challenges and perspectives toward the therapeutic use of bioengineered HFs for hair loss disorder are also discussed.

## 1 Introduction

Mammalian skin appendages, which include hair follicles (HFs), sweat glands, sebaceous glands, and nails, are derived from ectodermal epithelial and mesenchymal compartments of the skin (Mikkola, 2007; Arda et al., 2014). Among these, the HFs are one of the most complex structures and are considered a self-renewed miniorgan that is involved in physical protection, sensation, thermoregulation, and wound healing (Schneider et al., 2009; Lim et al., 2019; Lin et al., 2022). The absence of hair intensely impacts both physical and psychological well-being as it influences social interactions (Paus and Cotsarelis, 1999; Schneider et al., 2009). Therefore, hair loss treatment is in high demand. Several treatment modalities, such as drugs, low-level laser light therapy, and hair transplantation, have been used to improve or decelerate the progression of androgenetic alopecia, a most common type of progressive hair loss (Adil and Godwin, 2017; Lolli et al., 2017; Jimenez et al., 2021). However, non-surgical treatment shows only modest outcomes, whereas scarcity of donor HFs hampers surgical treatment in patients with extensive baldness. Therefore, attempts have been made to develop novel treatment options for hair loss patients.

One of the most anticipated therapeutics is *de novo* HF regeneration, which may provide an unlimited source of HFs for transplantation. Stem/progenitor cells within HFs are known to contribute to the self-renewal ability of HFs and have been expected to be a candidate cell source for HF regeneration. In addition, induced pluripotent stem cells (iPSCs), which are generated by reprogramming various types of somatic cells by defined factors, have been used to generate multiple cell types including cells of the HFs. In this review, we provide basic knowledge of HF morphogenesis, HF cycling, and HF regeneration from follicular cell sources and pluripotent stem cells (PSCs). In addition, challenges and future directions for the clinical use of regenerated HFs are described.

## 2 Hair follicle morphogenesis and cycling

**FIGURE 1:**
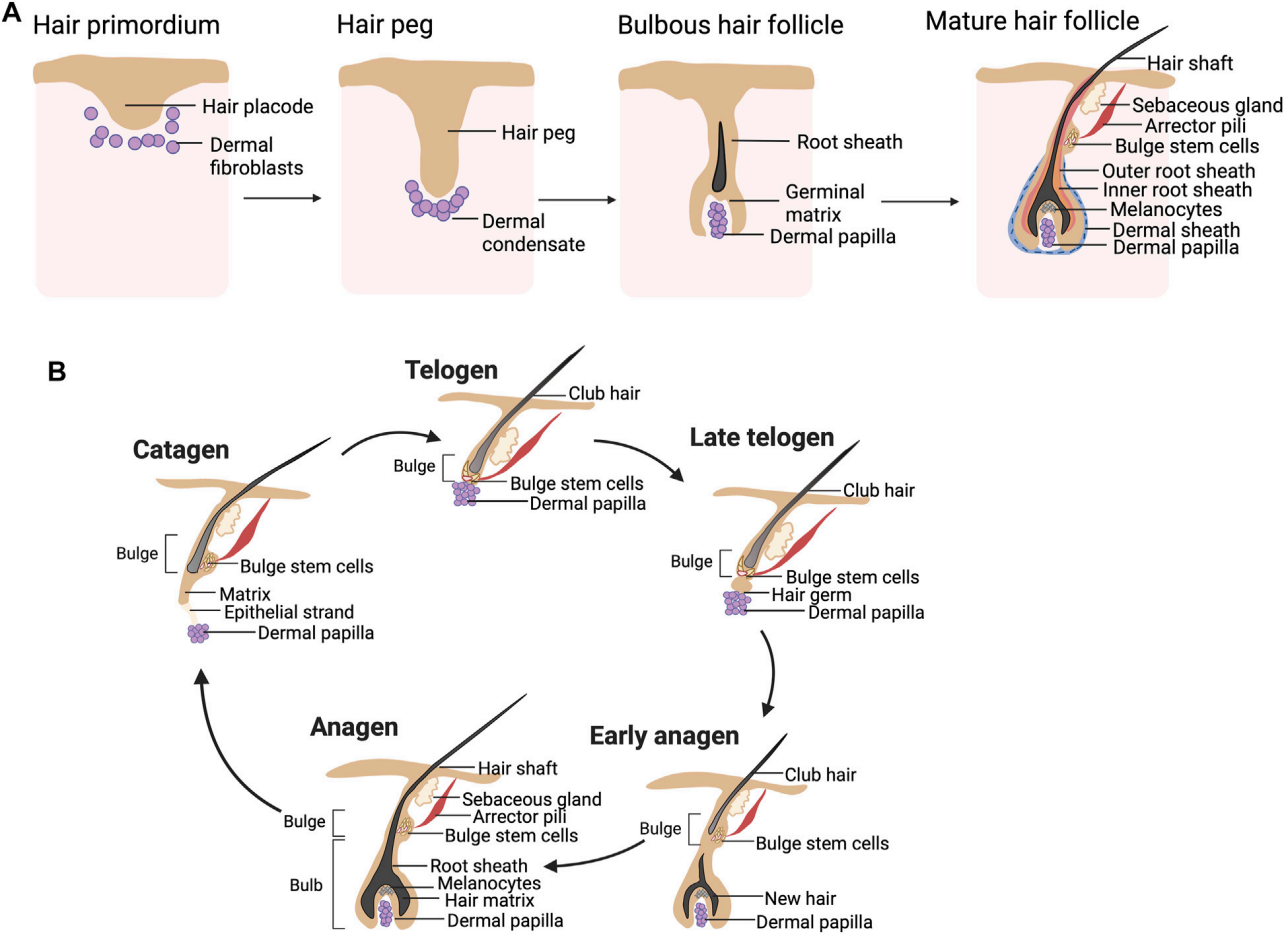
**(A)** Hair follicle morphogenesis. An interaction between the hair placode and underlying mesenchymal cells results in the formation of a hair peg, which has a proximal end surrounded by dermal condensate. The formation of the hair shaft, inner root sheath (IRS), and outer root sheath (ORS) is found in the bulbous hair peg. The mature hair follicles (HFs) consist of two main compartments: the epithelial compartment (hair matrix, IRS, and ORS including the bulge) and the dermal compartment (dermal papilla and dermal sheath). **(B)** Hair cycle. The hair shaft is growing throughout the anagen. During catagen, massive cell death is found in the lower part of HFs, while the bulge stem cells are preserved. The DP is migrating to have close contact with the quiescent bulge stem cells during telogen. During the telogen-to-anagen transition, the hair germ is formed and initiates new hair shaft generation.

Approximately 5 million HFs are present in human neonates (Paus and Cotsarelis, 1999). These HFs undergo repetitive hair cycles to regenerate themselves throughout life. The development of HFs initiates during the third month of pregnancy via the interaction between the epidermis and underlying mesenchymal cells. Wnt and bone morphogenetic proteins (BMPs) dictate the epidermal fate acquisition of ectodermal cells (Fuchs, 2007) while underlying mesenchymal cells of the dermis require Wnt signaling for their specification (Atit et al., 2006). The mesenchymal cells then induce the overlying epithelium to proliferate downward into the dermis and form hair placodes (Figure 1A) (Fuchs, 2007; Schneider et al., 2009). The formation of dermal condensate, a primordial structure of the dermal papilla (DP), was facilitated by the placode (St-Jacques et al., 1998). The dermal condensates influence the placode to grow more downward, becoming a hair germ and then a hair peg (Figure 1A). The bulbous hair peg represents a stage where the hair shaft, inner root sheath (IRS), and outer root sheath (ORS) are formed (Schneider et al., 2009).

The epithelial compartment of the mature HFs (Figure 1A) consists of matrix keratinocytes, IRS, and ORS. The matrix keratinocytes are actively proliferating cells located in the hair bulb and contribute to the hair shaft. Pigmentation of the growing hair shaft is regulated by melanocytes located adjacent to the matrix cell. The IRS is a group of keratinized cells that encases the matrix keratinocytes and controls the shaping of the hair shaft (Paus and Cotsarelis, 1999). The keratinized ORS covers all epithelial compartments. The bulge is located in the upper ORS at the insertion of the arrector pili muscle and has been known to contain HF stem cells (HFSCs), replenishing epithelial cells of HFs (Paus and Cotsarelis, 1999). The mesenchymal compartment is separated from the epithelial portion of the HFs by the basement membrane and is divided into the DP and dermal sheath (DS) (Ohyama et al., 2010; Yang and Cotsarelis, 2010). The DP is located in the hair bulb and plays an important role in hair cycling, hair shaft generation, and hair pigmentation (Morgan, 2014). The DS (also known as connective tissue sheath) is a structure line epithelial component of the HFs and is known to contain dermal sheath cup cells (DSCs), a precursor of DP cells (DPCs) (Yang and Cotsarelis, 2010).

Adult HFs are regularly capable of regenerating themselves through the hair cycle. Three phases of the hair cycle, namely, anagen, catagen, and telogen, govern the growth and shedding of the hair shaft (Figure 1B). During anagen, which could last for up to 6 years, the hair shaft continues growing, making the anagen HFs predominate in the human scalp (Castro and Logarinho, 2020; Ohyama, 2021). The HFs enter catagen when follicular keratinocytes within the matrix, IRS and ORS, as well as melanocytes, undergo apoptosis, while the bulge HFSCs are spared (Paus and Cotsarelis, 1999; Stenn and Paus, 2001; Schneider et al., 2009). As a result, the DP at the hair bulb is separated from the old hair shaft (club hair). Catagen usually lasts for 2–3 weeks (Ohyama, 2021). At the transition between catagen and telogen, the DP is pushed upward to make direct contact with the bulge. During the 2-month period of telogen, the club hair remains in the hair orifice but can be easily shed from the HFs. It is estimated that 50–150 club hair is lost per day (Paus and Cotsarelis, 1999). The bulge of telogen HFs is quiescent, hence the naming of telogen as a resting phase. Fibroblast growth factor 7 (FGF7) derived from the DP promotes telogen to anagen transition (Greco et al., 2009). During the early anagen of the next cycle, the DP sends these signals to activate quiescent bulge stem cells to proliferate, migrate downward, and differentiate into follicular keratinocytes.

## 3 Hair follicle regeneration from follicular cell sources

### 3.1 Dermal papilla cells and dermal sheath cup cells

**FIGURE 2:**
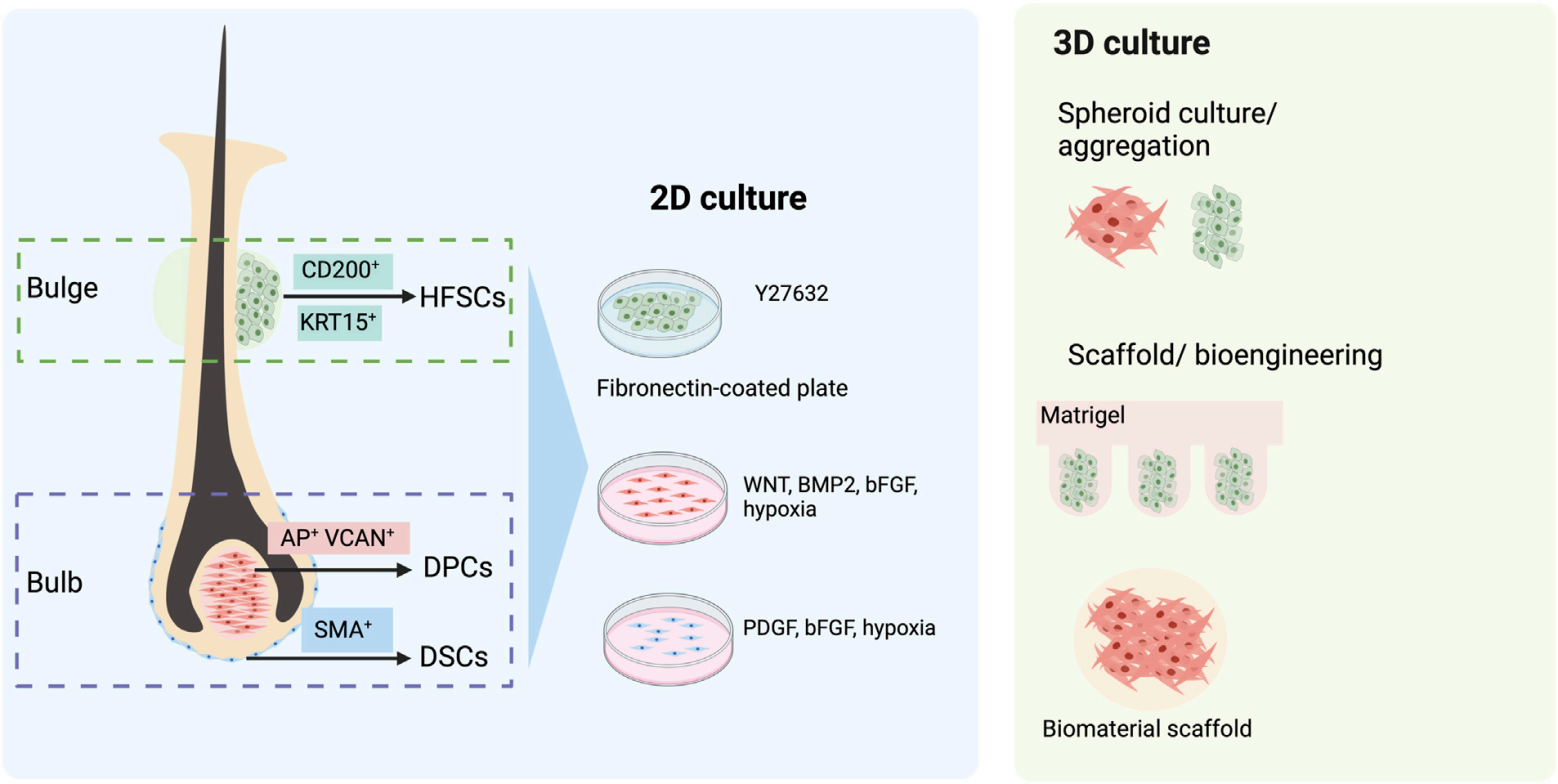
Follicular cell sources for hair follicle (HF) regeneration. The dermal compartment of HF comprises dermal papilla cells (DPCs) and dermal sheath cup cells (DSCs), which can be identified by their specific markers, alkaline phosphatase (AP), versican (VCAN), and smooth muscle actin (SMA). When isolated and expanded in 2D culture, their hair-inductive capacity could be maintained by the addition of growth factors such as Wnt, BMP2, bFGF, and PDGF. In addition, the hypoxic condition has been shown to augment the proliferation and trichogenicity of these cells. Alternatively, a 3D culture of DPCs has been demonstrated to improve hair-inductive ability. The epidermal compartment of HFs that can be used for HF bioengineering is CD200-positive, K15-positive hair follicle stem cells (HFSCs). The maintenance of HFSCs *in vitro* can be assisted by the use of a keratinocyte serum-free medium with Y-27632 and a fibronectin-coated culture dish. The 3D culture of HFSC aggregates in Matrigel also augments their proliferation.

Within HFs, the dermal compartment can be divided into DP and DS. DPCs are the key components of HF growth and can be identified by functional molecules such as alkaline phosphatase (AP) or versican (VCAN, Figure 2) (Ohyama and Veraitch, 2013; Ji et al., 2021). It is known that DPCs of the cephalic part originate from the neural crest (Wong et al., 2006), whereas DPCs from other parts of the body are derived from the mesoderm (Dequeant and Pourquie, 2008). An ability to induce hair formation (trichogenicity) of cells obtained from the DP has long been demonstrated (Oliver, 1967; 1970; Reynolds et al., 1999; Ohyama et al., 2010; Yang and Cotsarelis, 2010). Both freshly isolated and cultured DPCs influence neighbor epithelium HFSCs through a complex cascade of signaling pathways, further promoting the induction of the non-hair-bearing epidermis to form HFs (Higgins et al., 2013). Thus, DPCs, if expanded *in vitro*, would be an ideal source of dermal cells for HF regeneration. Nevertheless, two main issues hamper the use of cultured DPCs. First, the isolation of human DPCs from HFs by microdissection is burdensome and yields a limited number of cells. Second, it has been known that, when DPCs are cultured *in vitro*, they often lose their hair inductivity property (Jahoda et al., 1984; Horne et al., 1986; Osada et al., 2007). Further studies revealed an alteration in the transcriptional signature of DPCs cultured in a 2D environment as a cause of this loss and suggested that 3D spheroid culture generated by using the hanging drop method as well as 3D culture with the biomaterial scaffold could partially restore inductivity (Figure 2) (Higgins et al., 2013; Betriu et al., 2020). Interestingly, increasing the size of the 3D spheroid by culturing DPCs on hydrophilic polyvinyl alcohol (PVA)-coated culture vessels improved trichogenic potential (Huang et al., 2013). Additionally, other approaches have also been developed to maintain the trichogenicity of DPCs. Supplementation of cytokines involved in Wnt, BMP, and FGF signaling resulted in improving and stabilizing hair induction capacity in cultured DPCs (Figure 2) (Ohyama et al., 2012). Likewise, when cultured under a hypoxic condition (2% O_2_), trichogenicity and proliferation of DPCs were enhanced (Zheng et al., 2019).

It has been proposed that the DS may contain a subpopulation of stem cells replenishing the cells of the dermal compartment of HFs. Indeed, HF dermal stem cells (hfDSCs), a population of cells in the DS that exhibits self-renewal properties and the ability to repopulate cells of DS and DP, have been identified (Rahmani et al., 2014). Similar to DPCs, the hair-inductive capacity of DSCs has been demonstrated. *De novo* folliculogenesis was found in rats after co-implantation of DSCs and ORS epidermal cells into small wounds (Reynolds and Jahoda, 1996). When transplanted into ears and footpads, mouse DSCs were able to induce new HFs (McElwee et al., 2003). Interestingly, HF regeneration was found after the implantation of human DSCs from a male donor into a forearm of a female recipient (Reynolds et al., 1999). A recent clinical study on patients with male and female pattern hair loss also demonstrated that autologous injection of 300,000 DSCs increased hair density (Tsuboi et al., 2020). Nevertheless, the use of DSCs for HF regeneration also encounters limitations similar to DPCs, and several solutions have been proposed to overcome these problems. The maintenance of DSCs in cultures with the platelet-derived growth factor (PDGF) has been shown to enhance their hair inductivity and proliferation (Figure 2) (Gonzalez et al., 2017). An enhancer of the Wnt signaling pathway, R-spondin, stimulates DSC expansion (Hagner et al., 2020). Additionally, augmentation of proliferation and hair-inductive ability was reported in DSCs cultured under a hypoxic condition (1% O_2_) (Kanayama et al., 2020). Altogether, the therapeutic potential of DPCs and DSCs in hair disorders is unquestionable, and the development of a robust platform that could augment DPC and DSC expansion and maintain their trichogenicity is demanded.

### 3.2 Hair follicle stem cells

HFSCs are a group of heterogeneous cells located in the bulge, a permanent portion of the ORS (Inoue et al., 2009). Despite being able to rapidly proliferate during early anagen or wound healing, their salient characteristic is being quiescent throughout most of their lifespan (Ma et al., 2004; Cotsarelis, 2006). Human HFSCs are characterized by the expression of several markers, such as keratin 15 (KRT15), keratin 19 (KRT19), and CD200 (Figure 2) (Ohyama et al., 2006; Inoue et al., 2009; Purba et al., 2014; Wen et al., 2021). HFSCs exhibit a wide range of capacities to differentiate, not only into all cell types within the epithelial compartment of HFs, epidermis, and sebaceous gland (Blanpain et al., 2004; Ma et al., 2004; Morris et al., 2004; Cotsarelis, 2006; Kiani et al., 2018) but also into mesenchymal lineages under a suitable environment (Xu et al., 2013; Cheng et al., 2020). Clinical data on the use of HFSCs in alopecia areata (AA) and androgenetic alopecia (AGA) have recently been reported (Elmaadawi et al., 2018). The HFSCs were expanded *in vitro* before being autologously injected (100,000 cells per 1 cm^2^ injection site) into bald scalps. Clinical assessment performed at 3 and 6 months after the treatment showed an improvement in both AA and AGA patients receiving HFSC injections.

Although HFSC characteristics could be significant in regenerative medicine, the cultivation of HFSC *in vitro* caused a rapid loss of their stem cell abilities and triggered spontaneous differentiation (Blanpain et al., 2004; Ji et al., 2021; Wen et al., 2021). Attempts have been made to develop a culture system that would allow long-term maintenance of HFSCs. Three-dimensional culture with basic FGF (bFGF), vascular endothelial growth factor A (VEGF-A), and Y-27632 supplementation has been shown to preserve self-renewing capacity and multipotency of murine HFSCs (Chacon-Martinez et al., 2017). Interestingly, the xeno-free culture of human HFSCs using the keratinocyte serum-free medium supplemented with Y-27632 on fibronectin-coated culture vessels has recently been demonstrated to stimulate proliferation, maintain stemness, and enhance the ability to regenerate HFs of HFSCs (Figure 2) (Wen et al., 2021). In a recent study, both mouse and human HFSCs could be expanded by culturing the cells in uniform-diameter aggregates and encapsulating them in Matrigel (Hirano et al., 2023). Further development for high-throughput long-term HFSC culture would be essential for HF bioengineering.

## 4 Hair follicle regeneration from pluripotent stem cells

Due to their self-renewal and differentiation ability toward any cells derived from three embryonic germ layers, PSCs are considered invaluable cell sources for HF bioengineering. Embryonic stem cells (ESCs) have long been used as a major PSC source for generating various types of cells including the cells of HFs (Gnedeva et al., 2015). Nevertheless, the main hindrances of ESCs are immunological rejection and ethical consideration. Recently, reprograming somatic cells into iPSCs by forced expression of specific factors has emerged as an alternative strategy for stem cell-based applications (Takahashi et al., 2007; Yu et al., 2007). Despite having similar characteristics of self-renewal and differentiation ability to ESCs, iPSCs can be used autologously and are devoid of ethical concerns. Thus, iPSCs have been expected as a promising cell source for HF bioengineering, providing that robust differentiation of iPSCs toward folliculogenic cells is available.

A traditional approach for generating any specific somatic cell types from iPSCs is to differentiate iPSCs using optimized protocols. Nevertheless, most organs including HFs comprise multiple cell types. Generation of such complex structures, thus, requires induction of individual cell types from iPSCs, in this case, trichogenic dermal and folliculogenic epidermal cells, followed by assembling them in an environment permitting 3D organization of these cells. Indeed, the aforementioned approach is widely used in HF regeneration although multiple steps involved may be considered a limitation (Ohyama, 2021). In another approach, HFs can be induced from iPSCs by self-organization of differentiating iPSCs into 3D structures such as organoids (Lee et al., 2020). The latter approach involves fewer steps, and the generated HFs would resemble more *bona fide* HFs, albeit some limitations such as batch-to-batch variation do exist (Sun et al., 2021).

### 4.1 Generation of individual HF components

#### 4.1.1 iPSC-derived trichogenic dermal cells

**FIGURE 3:**
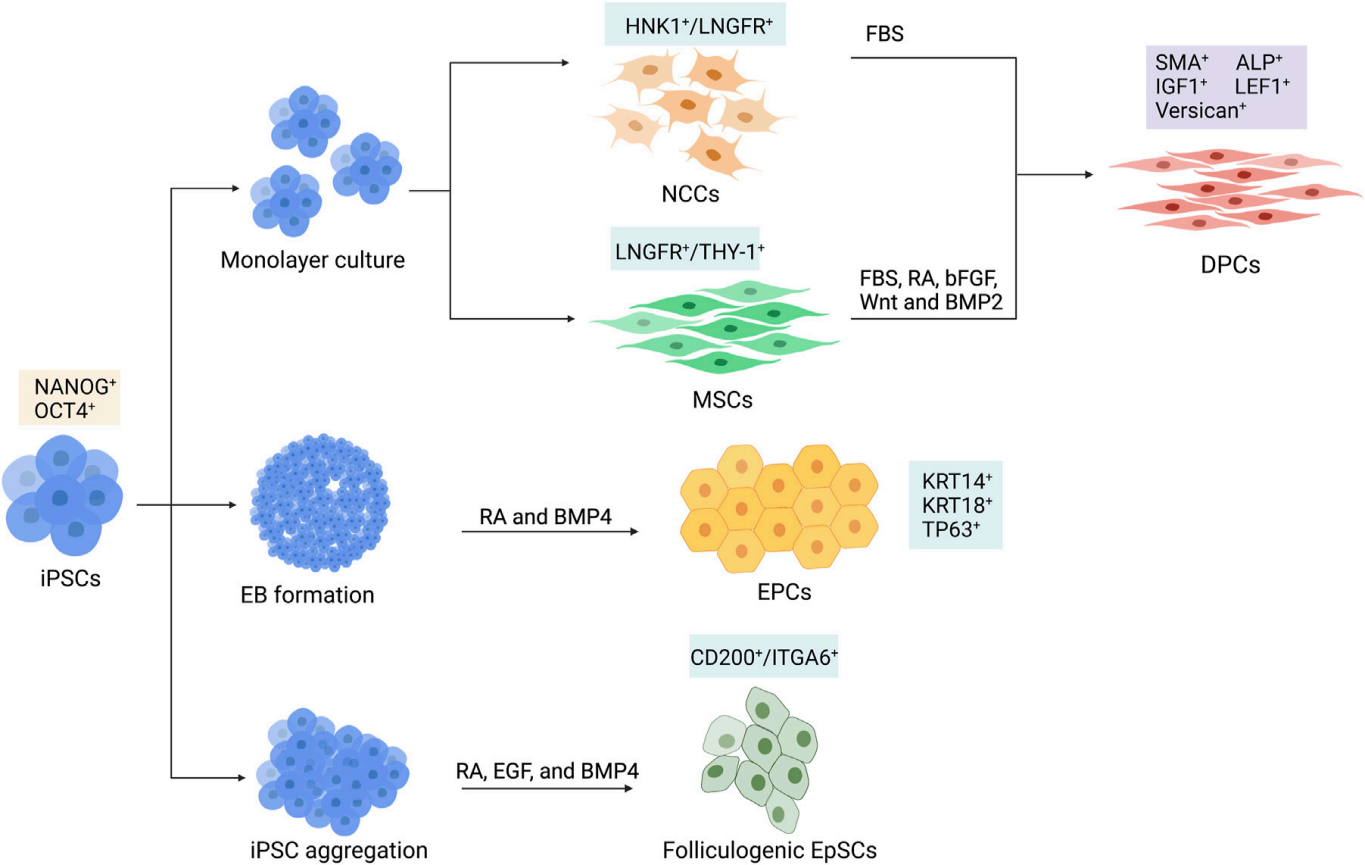
Schematic differentiation of human induced pluripotent stem cells (hiPSCs) toward dermal papilla cells (DPCs), ectodermal precursor cells (EPCs), and folliculogenic epithelial stem cells (EpSCs). DPCs can be generated by the differentiation of hiPSCs into intermediate cell types, which could be HNK1^+^/LNGFR^+^ neural crest cells (NCCs) or LNGFR^+^/THY-1^+^ mesenchymal stem cells (MSCs). hiPSC-derived NCCs are further differentiated into DPCs in culture supplemented with fetal bovine serum (FBS). Differentiation of MSC into DPC requires FBS, retinoic acid (RA), bFGF, Wnt, and BMP2. The KRT14^+^/KRT18^+^/TP63^+^ EPCs can be generated from hiPSC-derived embryoid bodies (EBs) by adding RA and BMP4 in the culture medium. Generation of CD200^+^/ITGA6^+^ EpSCs can be achieved by culturing hiPSC in aggregates with RA, epidermal growth factor (EGF), and BMP4.

The generation of dermal components with the ability to induce hair formation is required for HF bioengineering. Due to the limitations of DPC and DSC cultivation mentioned in the previous section, the iPSC-derived trichogenic dermal cells could be an alternative replacement. Data from several studies indicated that a portion of DPCs, including the cephalic DPCs, originated from neural crest cells (NCCs) (Le Douarin and Dupin, 1993; Fernandes et al., 2004; Nagoshi et al., 2008; Driskell et al., 2009). Thus, it would be possible to generate DPCs by differentiating hPSCs into NCCs and then direct differentiation of hPSC-derived NCCs into DPCs (Figure 3). Gnedeva et al. (2015) developed a protocol to generate hair-inducing hPSC-derived DP-like cells using this strategy (Table 1). NCCs were initially converted from hPSCs via neurosphere generation before plating the spheres on fibronectin-coated culture vessels. The generated NCCs expressed NCC signature markers such as LNGFR, SOX10, and FOXD3 and contained a population of cells that expressed mesenchymal stem cell markers such as CD47, CD184, and CD44 (Gnedeva et al., 2015). Induction of DPCs was performed by transferring the dissociated hPSC-derived NCCs to non-coated tissue culture plastic with serum-containing DP medium. The adherent cells were maintained for 2 weeks and expressed DPC markers including LNGFR, versican, smooth muscle actin (SMA), and AP (Yang and Cotsarelis, 2010; Gnedeva et al., 2015). Co-transplantation of the hPSC-derived DPCs with embryonic murine epidermal cells into nude mice led to HF induction, demonstrating trichogenicity of the hPSC-derived DPCs. Furthermore, the authors reported that the hair-inductive ability of hPSC-derived DPCs was dependent on BMP signaling. Inhibition of BMP signaling by dorsomorphin during NCC to DPC differentiation resulted in morphological alteration and loss of hair-inducing properties of the generated cells.

**TABLE 1:** Generation of DPCs and epithelial cells from hiPSCs.

Target cells/cell source	Medium composition	Supplemental factor	Culture type	Markers presented	Key outcome	Reference
A) Dermal papilla cells						
hECSs (H9); hiPSCs	First step: DMEM/F12 and neurobasal (1:1)	First step: B27	First step: neurosphere formation in a low-adhesion dish then plated on a fibronectin-coated dish	P75, nexin-1, versican, SMA, vimentin	Induce HF formation when co-transplanted with mouse keratinocytes into nude mice	Gnedeva et al. (2015)
	Second step: DMEM/F12	Second step: FBS	Second step: adherent culture using an uncoated dish			
hiPSCs	First step: StemPro MSC-SFM CTS	First step: -	First step: embryoid body formation then plated on a CELLstart CTS-coated dish	ALP, LEF1, WNT5A, IGF1	Improve DPC trichogenicity by co-culture with human keratinocytes; hair fiber generation when co-grafted with human keratinocytes	Veraitch et al. (2017)
	Second step: DMEM	Second step: FBS and RA then FBS, BIO, BMP2, and bFGF	Second step: adherent culture using an uncoated dish			
B) Epithelial cells						
hiPSCs	D-KSFM (defined keratinocyte serum-free medium)	RA, BMP4	Embryoid body formation then plated on a collagen I-coated dish	KRT14. KRT18, TP63	Upregulation of follicular keratinocyte markers when co-cultured with human DPCs; HF formation when co-transplanted with mouse dermal cells into nude mice	Veraitch et al. (2013)
hiPSCs	DMEM/F12 (days 1–7)	Initially low BMP4, followed by RA, then RA + BMP4 + EGF, then BMP4 + EGF, and EGF alone	embryoid body formation followed by culture on 3T3 feeders and then under feeder-free conditions	CD200, ITGA6	Co-grafting CD200^+^/ITGA6^+^ EpSC with dermal cells in nude mice; patch assays	Yang et al. (2014)
	KSFM (keratinocyte serum-free medium, days 8–11)					
	KSFM + D-KSFM 1:1 (days 11–25)					

Recently, the protocol for generating DPCs via NCC intermediate has been further optimized by Pinto and Terskikh (2021). Instead of using an undefined component such as fetal bovine serum (FBS), they developed a more clinically suitable chemically defined medium composed of factors involved in HF development. It is well known that Wnt signaling plays an important role in HF morphogenesis (Andl et al., 2002). Likewise, FGF20 is reported to control the formation of dermal condensation (Huh et al., 2013), and BMP6 is required for the maintenance of hair-inducing ability (Rendl et al., 2008). For these reasons, a combination of Wnt10b, R-spondin, FGF20, and BMP6 was used to generate DPCs from a human-induced pluripotent stem cell (hiPSC)-derived NCCs. Furthermore, the hiPSC-derived DPCs generated from this protocol were able to induce HF formation when co-transplanted with human bulge epithelial stem cells into nude mice (Pinto and Terskikh, 2021).

It is known that, apart from the facial dermis, the majority of dermal cells derive from the mesoderm (Dequeant and Pourquie, 2008). Additionally, DPCs share some similarities in phenotype with mesenchymal cells. Thus, it is possible that DPCs may also arise from mesenchymal stem cells (MSCs) (Yang and Cotsarelis, 2010; Veraitch et al., 2017). Generation of hiPSC-derived DPCs could be performed by first differentiating hiPSC into mesenchymal intermediate and then instructing the hiPSC-derived mesenchymal cells toward DPCs (Figure 3). Veraitch et al. (2017) described the generation of induced mesenchymal cells (iMCs) expressing CD29, CD44, CD90, and CD166 from hiPSCs (Table 1). The iMCs were able to differentiate into osteoblasts, chondrocytes, and adipocytes, confirming their mesenchymal stem cell property. Interestingly, a population of iMCs with LNGFR and THY-1 expression could be purified and maintained on plastic culture vessels. When LNGFR^+^/THY-1^+^ iMCs were cultured in a serum-containing medium with RA supplementation before changing to a DPC-activating culture (DPAC) medium consisting of FBS, WNT activator 6-bromoindirubin-3′-oxime, BMP2, and bFGF, the iMCs acquired the DPC phenotype as demonstrated by an upregulation of DPC signature genes, such as *RGS2*, *BMP4*, *LEF1*, and *BAMBI*, as well as an increase in alkaline phosphatase activity. Moreover, the epithelial–mesenchymal interaction between the induced DP substituting cells (iDPSCs) generated from this protocol and human keratinocytes was demonstrated in a co-culture experiment. Subcutaneous transplantation of collagen gel containing iDPSCs and human keratinocytes covered with human fibroblasts into severe combined immunodeficient (SCID) mice led to hair shaft formation within the grafts, albeit at much less frequency compared to co-transplantation with human DPCs (Veraitch et al., 2017; Ohyama, 2019). These findings confirmed that the trichogenic DP-like cells can be generated from hiPSCs. However, further optimization of the differentiation protocol is required to increase conversion efficiency as well as to improve trichogenicity of the DPC equivalent cells.

#### 4.1.2 iPSC-derived folliculogenic epidermal cells

Since the epithelial part of HFs is derived from the epidermal stem cells that resided in the bulge area of the HFs (also known as HFSCs), the generation of such a population would be needed for HF regeneration. Interestingly, several protocols for generating epidermal stem cells and keratinocyte progenitor cells from hPSCs have been well established (Metallo et al., 2008; Guenou et al., 2009; Itoh et al., 2011; Tadeu and Horsley, 2013; Sebastiano et al., 2014; Sah et al., 2021). Despite a variation in the culture system (feeder-dependent vs. feeder-free, different types of culture media) used in each protocol, the use of retinoic acid (RA) and BMP4 in most protocols effectively promoted ectodermal fate acquisition (Metallo et al., 2008) and inhibited neural induction of PSCs (Gambaro et al., 2006), respectively. The iPSC-derived keratinocytes obtained from these protocols expressed keratin 14 (KRT14), indicating that they were mitotically active basal layer epidermal stem/progenitor cells (Alam et al., 2011). Although these interfollicular epidermal stem cells should be able to generate HFs when induced by DPCs, the HFSC population in the bulge of HFs was a main contributor to HF growth and cycling (Morris et al., 2004; Ito et al., 2005; Levy et al., 2005; Blanpain and Fuchs, 2006; Kaur, 2006). Thus, comprehensive characterization of hiPSC-derived epidermal cells obtained from the aforementioned studies, especially the expression of HF bulge markers CD200, ITGA6, and KRT15 (Ohyama et al., 2006), would be useful for determining the HFSC identity of these cells. In fact, another study reported that only a small proportion of cells derived in cultures with RA and BMP4 were CD200-positive and ITGA6-positive (Yang et al., 2014).

During a process of hiPSC differentiation toward mature keratinocytes, there might be some precursor cells that would be able to receive signals from hair-inductive dermal cells and contribute to HF formation. Indeed, Veraitch et al. (2013) reported the generation of ectodermal precursor cells (EPCs, Figure 3) from hiPSCs via embryoid body formation in the presence of RA and BMP4 (Table 1). The hiPSC-derived EPCs expressed keratin 18 (KRT18) and KRT14, indicating that they were epidermal progenitors. When co-cultured with human DPCs, one hiPSC-derived EPC line upregulated hair-associated genes *KRT75*, *MSX2*, *LEF1*, and *TRPS1*. Upregulation of DPC-related genes *ALPL*, *BMP4*, and *LEF1* was also observed in the co-cultured DPCs, indicating cross-talk between epithelial and mesenchymal components of HFs (Veraitch et al., 2013). Interestingly, co-injection of the hiPSC-derived EPCs and murine trichogenic neonatal dermal cells into the subcutaneous space of nude mice resulted in HF morphogenesis. HFs generated from co-injection of hiPSC-derived EPCs and mouse neonatal dermal cells were positive for human HFSC markers KRT15, CD200, and DIO2, while HFs generated from co-injection of mouse neonatal keratinocytes and dermal cells were negative, indicating that the hiPSC-derived EPCs could repopulate HFs, albeit at low efficiency. When undifferentiated hiPSCs and hiPSC-derived embryoid bodies were used instead of the hiPSC-derived EPCs, HF formation was also found. Nevertheless, the regenerated HFs did not exhibit human HFSC markers, indicating that these two types of human cells enhance HF regeneration via the non-cell autonomous mechanism rather than through the direct repopulation of HFs (Veraitch et al., 2013).

A study by Yang and colleagues demonstrated the generation of folliculogenic epithelial stem cells (EpSCs, Figure 3) expressing bulge stem cell markers CD200, ITGA6, and KRT15 from hiPSCs (Table 1) (Yang et al., 2014). Similar to previous studies, they initially utilized RA and BMP4 in their differentiation protocol and examined a population of cells expressed CD200 and ITGA6 during differentiation. They found that only a small fraction of cells (7.1%) was CD200^+^/ITGA6^+^. Interestingly, the addition of epidermal growth factor (EGF) into the culture was required for an enrichment (up to 26.8% at day 18 of differentiation) of the CD200^+^/ITGA6^+^ stem cell population (Yang et al., 2014). The hiPSC-derived EpSCs highly expressed epithelial lineage markers such as *KRT1*, *KRT8*, *KRT10*, *KRT15*, *ITGB1*, and *ITGA6* at levels similar to HF-derived EpSCs. Moreover, when performing skin reconstitution assay by injecting the hiPSC-derived CD200^+^/ITGA6^+^ EpSCs together with murine dermal cells subcutaneously into nude mice, HF-like structures with hair shafts, IRS, and ORS, as well as interfollicular epidermis, were generated within two and a half weeks. These findings confirmed that folliculogenic epidermal cells resembling bulge HFSCs can be obtained from the directed differentiation of hiPSCs.

#### 4.1.3 *In vitro* reconstruction of HFs with iPSC-derived dermal and epidermal cells

**FIGURE 4:**
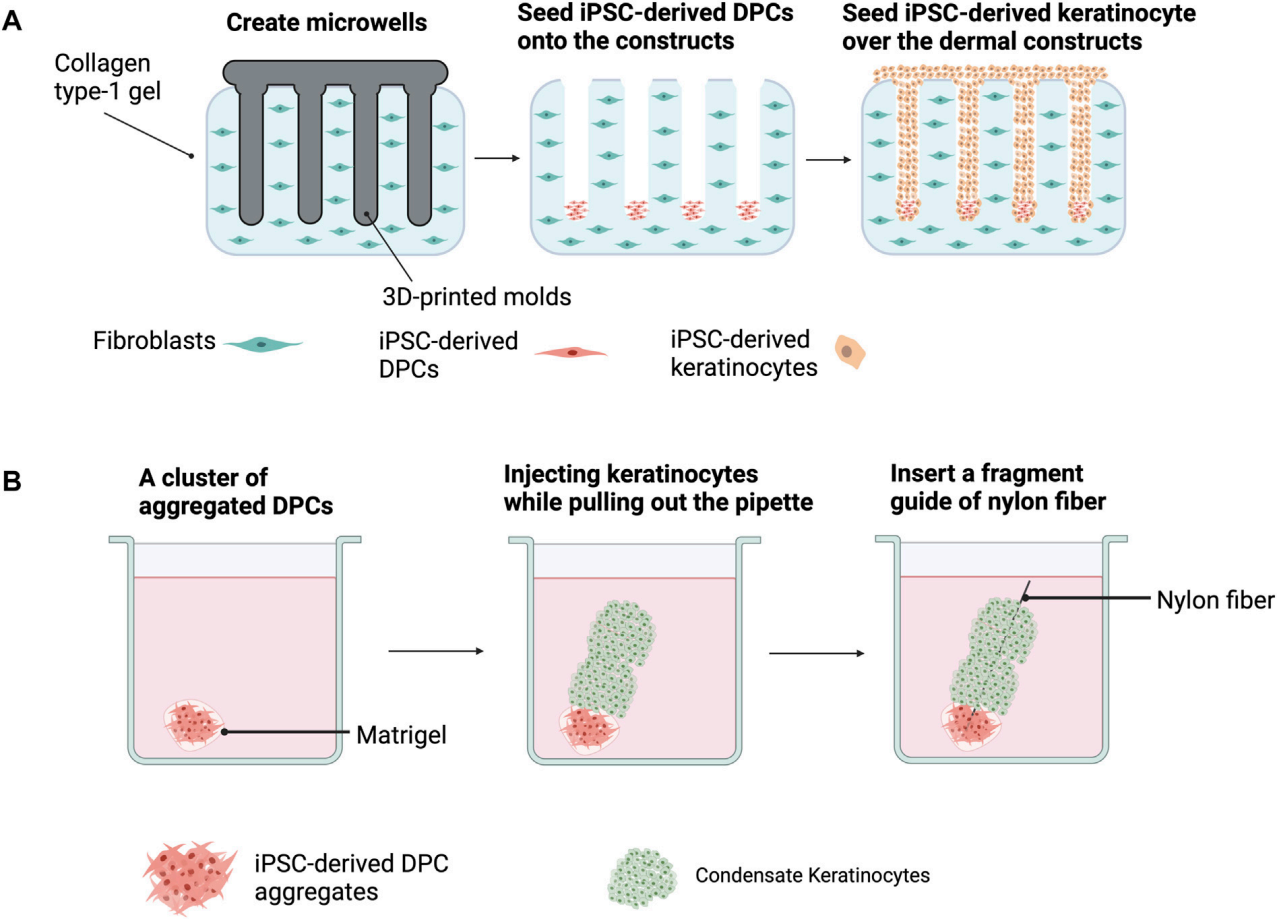
Hair follicle (HF) reconstruction from iPSC-derived dermal papilla cells (DPCs) and iPSC-derived keratinocytes using the biomimetic developmental approach **(A)** and 3D assemblage **(B)**. **(A)** 3D-printed molds are used to generate microwells on type 1 collagen gel containing dermal fibroblasts. DPCs are added into each microwell and DPC aggregates are spontaneously formed. The addition of keratinocytes into the gel results in HF formation. **(B)** HF regeneration by adding DPC aggregates into Matrigel before injecting condensed keratinocytes on top. A nylon fiber is inserted as a scaffold.

Most of the reconstitution assays for HF regeneration with hiPSC-derived trichogenic dermal cells and hiPSC-derived folliculogenic epithelial cells were performed *in vivo* using immunodeficient mice. However, regenerated HFs should be xeno-free for clinical utilization. Recently, Abaci et al. (2018) demonstrated that a 3D microenvironment allows the effective generation of HFs *in vitro* (Figure 4A). By using 3D printing technology, they created plastic molds with adjustable extensions and used them for generating microwells mimicking the HF shape on a type I collagen gel containing dermal fibroblasts. After seeding into the microwells, DPCs spontaneously formed aggregates at the bottom of these microwells. Keratinocytes were then added to the gel, allowing the creation of a 3D structure resembling HFs. Interestingly, the HF-like structures with cells expressing hair-specific markers and hair fibers were observed after 3 weeks of culture, confirming that functional HFs can be generated using the biomimetic developmental approach. Additionally, the reconstruction of HFs by the 3D assembly of human keratinocytes and hiPSC-derived DPC aggregates in the Matrigel matrix with a nylon wire guide as a scaffold has been reported (Figure 4B) (Fukuyama et al., 2021). Since trichogenic dermal cells, folliculogenic epidermal cells, and melanocytes (Ohta et al., 2011) can be generated from hiPSCs, reconstruction of pigmented HFs with hiPSC-derived cells using one of the aforementioned approaches could possibly be a crucial step for further applications (Ohyama, 2021).

### 4.2 Generation of entire HF from iPSCs

#### 4.2.1 3D IOS

**FIGURE 5:**
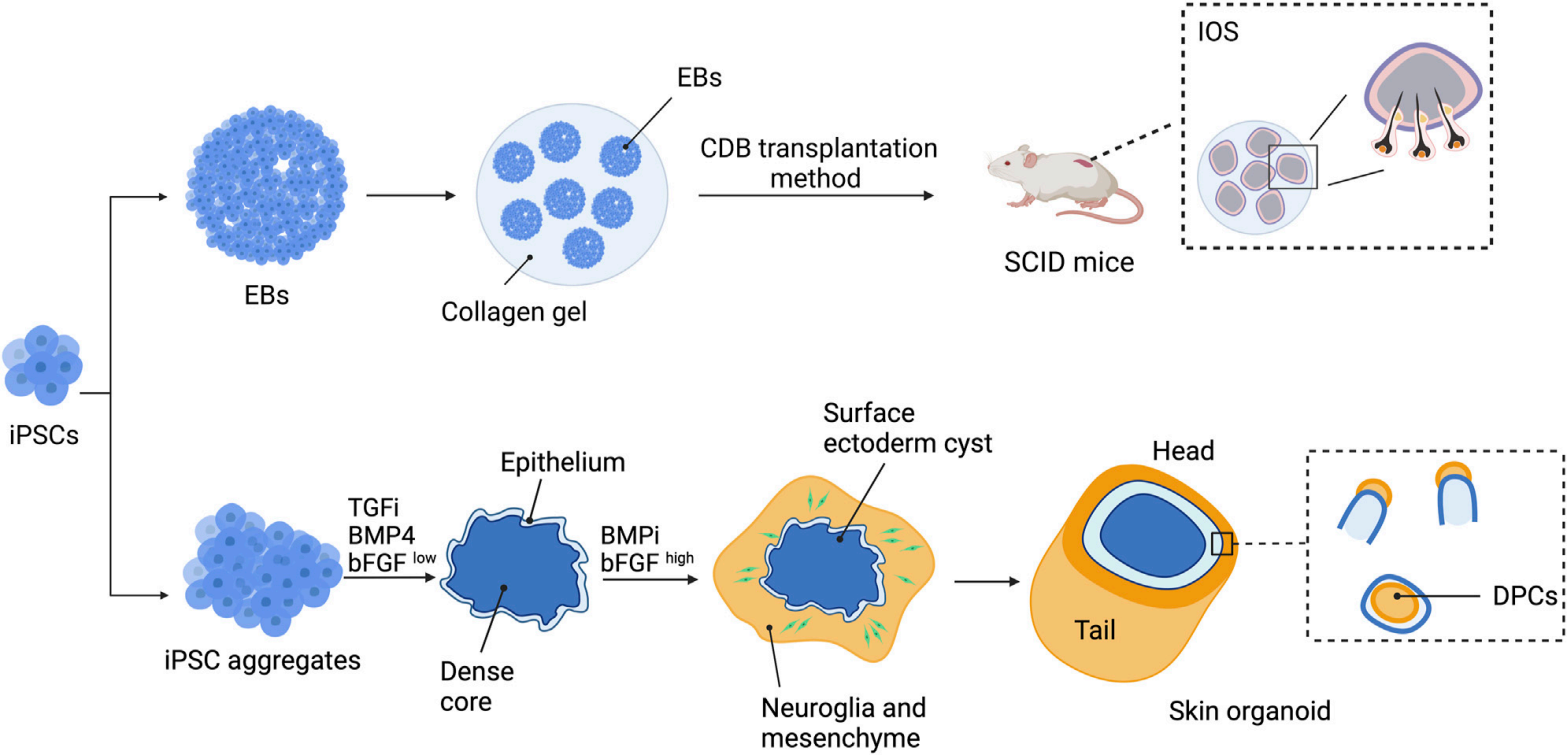
Strategies to generate bioengineered HFs using a 3D integumentary organ system (3D IOS) and skin organoid. 3D IOS is performed by generating embryoid bodies (EBs) and embedding the EBs in collagen gel. Clustering-dependent EB (CDB) transplantation is then performed by injecting collagen gel-embedded EBs into the subrenal capsule of severe combined immunodeficient (SCID) mice. Skin organoids are generated by treating hiPSC aggregates with BMP4, TGF inhibitor (TGFi), and low-concentration bFGF (bFGF^low^) for 3 days before changing to BMP inhibitor (BMPi) and high-concentration bFGF (bFGF^high^). The aggregates will develop into skin organoids with hair follicles and can be maintained for up to 150 days.

Tsuji et al. successfully generated a bioengineered 3D integumentary organ system (IOS) from mouse iPSCs (miPSCs) by using an *in vivo* clustering-dependent embryoid body (CDB) transplantation method (Figure 5) (Takagi et al., 2016; Toyoshima et al., 2019). In brief, embryoid body formation of miPSCs in the presence of Wnt10a was used for allowing differentiation toward epithelial and mesenchymal fate. Transplantation of multiple EBs (>30) embedded in collagen gel into the subrenal capsule of SCID mice was then performed to allow *in vivo* induction of bioengineered 3D IOS consisting of skin, HFs, sebaceous glands, and subcutaneous adipose tissue. The generated HFs were abundant and contained mature HF cells and structures such as bulge stem cells, melanocytes, DP, and DS (Takagi et al., 2016). Moreover, when isolated from the explant and re-transplanted into nude mice, the miPSC-derived 3D IOS could engraft, connect properly to the surrounding tissues of the recipient, and generate a black hair shaft. Additionally, bioengineered HFs did not show significant differences in hair types and distribution compared with natural hair of adult murine (Takagi et al., 2016). Nevertheless, whether a similar approach is reproducible in hiPSCs has yet to be proven. In addition, the development of an *in vitro* system that allows human 3D-IOS production is required for generating bioengineered HFs for clinical utilization.

#### 4.2.2 Skin organoid

Derivation of the skin organoid consisting of the epidermal layer, dermal layer, and skin appendages from mouse PSCs (mPSCs) has been reported by Lee et al. (2018). mPSC aggregates were initially treated with TGF-β inhibitors SB431542 and BMP4 followed by bFGF and the BMP inhibitor LDN-193189 to induce the organization of the organoid. By day 8 of induction, the aggregates consisted of three different populations: the outer surface differentiated into epithelial cells of the surface ectoderm, the intermediate layer contained mesodermal and neuroectodermal cells, and the innermost area contained undifferentiated mPSCs. The developing skin organoids then underwent inside-out transformation, in which the intermediate layer protruded and covered the surface ectoderm. Within 30 days of differentiation, the skin organoids consisted of the stratified epidermis, dermis, hypodermis, HFs, and sebaceous glands. Using a similar approach with some modification (Figure 5), self-organized skin organoids from hESCs and hiPSCs were generated by the same research group (Lee et al., 2020; Lee et al., 2022). Induction of epithelial cysts occurred on day 8 of differentiation, and the cysts were covered by neuromesenchymal cells a week later. The hair placodes, germs, and pegs were detected at approximately days 55–75 of differentiation. Maturation of hair pegs to HFs, some with pigmentation, was found between days 70 and 130. The HFs within the skin organoids exhibited almost all HF structures except for the medulla layer, indicating that they were lanugo rather than terminal hair. Furthermore, neural innervation of the upper bulge of HFs within the skin organoids was observed. Single-cell RNA sequencing revealed that the hPSC-derived skin organoids were comparable to the second-trimester human fetal facial skin. The skin organoids were able to be maintained in culture for up to 150 days and underwent cystic-to-planar transition with the hair shaft growing out in the correct direction when implanted into the skin of nude mice.

## 5 Human follicular cells vs. hiPSCs for HF regeneration

Although human HFs can be regenerated from either follicular cell sources or hiPSCs, these two sources of cells have their own unique features that one should take into consideration before opting to use each approach for further applications (Table 2). Isolation of DPCs, DSCs, and HFSCs is known to be arduous with only an inadequate number of cells obtained from the process. Limited expansion capacity of these follicular cells *in vitro* and a loss of their phenotype essential for hair induction during culture are the main obstacles for subsequent utilization of this approach. On the other hand, hiPSCs have unlimited self-renewal capacity, minimizing the problem of cell shortage. Preservation of hair-inductive/receptive abilities of the hiPSC-derived dermal and epidermal cells has been demonstrated (Yang et al., 2014; Gnedeva et al., 2015; Veraitch et al., 2017). Nevertheless, the downsides of the hiPSC-based approach should not be overlooked. Differentiation of hiPSCs toward trichogenic dermal cells and/or folliculogenic epidermal cells would take more time than isolation and expansion of these cells from HFs. In addition, tumor formation from contaminated hiPSCs that remained in culture is possible when using any hiPSC-derived products for cell-based therapy.

**TABLE 2:** Differences between two sources of cells, follicular cells, and hiPSCs, used for human HF regeneration.

	Human follicular cell	hiPSC
Starting materials	Scarce	Abundant
Expansion	Limited	Unlimited
Intrinsic property (e.g., hair-inductive ability)	Can be absent	Present
Risk of tumorigenesis	Lower	Higher
Ability to form skin organoid	Yes	Yes
Type of hair generated	Terminal hair	Lanugo or terminal hair
Normal hair cycling in regenerated HFs	No data	No data
Clinical utilization	Yes	No

In terms of the phenotype of regenerated HFs, it is preferred if these HFs are able to generate mature medullated terminal hair with long-term hair cycling ability. Although most studies have not investigated types of hair generated from follicular cell-derived HFs and hPSC-derived HFs, the generation of terminal hair from both types of regenerated HFs has been reported (Reynolds and Jahoda, 1996; Yang et al., 2014). The most challenging characteristic to be demonstrated in the bioengineered HFs is the ability to have normal hair cycling since this requires long-term maintenance of the regenerated human HFs, which could be years in the case of human terminal hair (Yang et al., 2014). To date, no study has depicted this issue in human HFs regenerated from both follicular cell sources and hPSCs. The development of culture and/or xenograft systems suitable for the long-term maintenance of bioengineered HFs may be useful for addressing the aforementioned issue.

## 6 Challenges and future perspectives

Despite tremendous improvement in HF reconstruction using hiPSCs, several issues need to be addressed before clinical utilization. First and foremost, safety issues on the use of hiPSCs must be taken into consideration. For clinical application, hiPSCs must be generated and characterized in compliance with current good manufacturing practice (cGMP) regulations (Rehakova et al., 2020). Reprogramming with integration-free methods is preferred to avoid unintentional mutagenesis and persistent transgene expression. Clinical-grade hiPSCs should be derived and maintained under xeno-free conditions to minimize the risk of recipients developing immune reactions against animal products (Wang et al., 2015). Critical quality attributes (CQAs) including identity determination, microbiological testing, genetic fidelity and stability testing, viability, and characterization of pluripotency properties are mandatory for clinical-grade hiPSC lines (Sullivan et al., 2018; Rehakova et al., 2020). Due to these requirements, it has been estimated that the entire process of manufacturing clinical-grade hiPSCs could be as long as 6–9 months and cost approximately 800,000 US dollars per line (Bravery, 2015; Huang et al., 2019). Hence, instead of using autologous hiPSCs, HLA-matched allogeneic hiPSCs or universal hiPSCs have been proposed to be an alternative and a more appropriate cell source for regenerative medicine (Umekage et al., 2019; Flahou et al., 2021).

Quality controls of the interested products, hiPSC-derived trichogenic dermal cells, hiPSC-derived folliculogenic epidermal cells, and hiPSC-derived skin organoids, are also essential. Most of the differentiation protocols for deriving trichogenic dermal cells and folliculogenic epidermal cells from hiPSCs are neither effective nor xeno-free. Optimization of these culture systems is needed to enhance the differentiation to have a more homogeneous population of interested cell types. The development of a chemically defined medium to be used instead of the serum-containing medium is also recommended. Additionally, morphological and molecular characterization of the hiPSC-derived dermal cells and hiPSC-derived epidermal cells must be performed to ensure that they phenotypically resemble their *bona fide* counterpart. Moreover, functional analysis of these cells is essentially required. Although the contribution to HF regeneration has been demonstrated in both hiPSC-derived dermal cells and hiPSC-derived epidermal cells, regeneration of functional human HFs with long-term hair cycling has not been reported. Unlike mouse HFs, demonstrating hair cycling in bioengineered human HFs is a challenging task since each hair cycle lasts from months in lanugo hair to 2–8 years in terminal hair (Yang et al., 2014; Houschyar et al., 2020; Lee et al., 2022). Thus, the development of a platform that could accommodate the long-term maintenance of regenerated human HFs is required. Alternatively, the identification of signaling cues that could shorten the hair cycle of human HFs may provide a chance to test whether hair cycling does exist in regenerated HFs. Furthermore, data from mouse studies demonstrated heterogeneity among HFSC populations and suggested that these subpopulations may have distinct functions (Joost et al., 2016; Chovatiya et al., 2021). Indeed, a recent study revealed a subpopulation of murine HFSCs expressing CD34, CD49f, and Itgβ5 is responsible for the long-term hair cycling of bioengineered HFs (Takeo et al., 2021). Although human bulge HFSCs do not express CD34 (Ohyama et al., 2006), it is of interest to investigate whether this analogous subpopulation of HFSCs exists and is involved in long-term hair cycling in humans. If so, identification of this subpopulation in hiPSC-derived HFSCs would be required to ensure that the reconstructed human HFs are able to have long-term hair cycling.

It is generally known that most of the hiPSC-derived cells are immature in phenotype (Doss and Sachinidis, 2019; Wiegand and Banerjee, 2019). Each human HF is able to generate three different types of hair, namely, lanugo, vellus, and terminal, depending on the person’s age and sex, and on the location of HFs (Blume-Peytavi and Vogt, 2011). In terms of further applications, the generation of mature pigmented medullated terminal hair, which is found primarily on the scalp, eyelashes, and eyebrows, would be preferable to immature lanugo and vellus hair, which lacks the medullary layer, has a thinner diameter, and is shorter in length. Interestingly, the type of hair generated from reconstructed human HFs can be varied. HF-like structures generated from a chamber-based skin reconstitution assay with hiPSC-derived EpSCs and neonatal mouse dermal cells were able to produce a terminal hair shaft with the medullary layer (Yang et al., 2014). On the other hand, the hiPSC-derived skin organoids produce lanugo-like hair, which lacks the medullary layer within the hair shaft, analogous to what is found in the human fetus in the second trimester of pregnancy (Langbein et al., 2010; Lee et al., 2020; Lee et al., 2022). Thus, it would be possible that the regenerated HFs could give rise to terminal hair, providing that proper signaling cues are present in their microenvironment.

Another concern relating to the use of hiPSC-derived products is the purity of interested cell types. It is very likely that, even if the differentiation protocols have been substantially optimized, contamination of hiPSCs or differentiated cells with undesired phenotypes may still persist. Thus, the elimination of undifferentiated iPSCs that remained in cultures is required to reduce the risk of tumorigenesis from unintentional transplantation of these cells into patients. This could be achieved by engineering an inducible suicide gene such as inducible caspase-9 into the iPSCs (Liu et al., 2022; Wunderlich et al., 2022). Such a system allows the undifferentiated iPSCs to undergo apoptosis in the presence of a chemical inducer of dimerization (CID) and diminishes the chance of developing teratomas from contaminated iPSCs. Alternatively, purification of cell types of interest may be performed to exclude the undifferentiated iPSCs and/or other undesirable differentiated cells. Several approaches have been used to purify interested cell types such as manual selection, enzymatic separation, gradient centrifugation, cell type-specific reporter, and flow cytometry-based cell sorting (Miki et al., 2015; Regha et al., 2022). Recently, microRNA-responsive, synthetic modified mRNA switch (miR switch), in which the synthetically modified mRNA was designed to contain the miRNA target sequence complimentary to specific miRNA and fluorescent reporter sequence, was used to purify hPSC-derived cardiomyocytes, endothelial cells, hepatocytes, and insulin-producing cells from undifferentiated hPSCs and other differentiated cells (Miki et al., 2015). Interestingly, large-scale purification of hiPSC-derived cells such as cardiomyocytes and pancreatic insulin-producing cells has been accomplished by a combination of the miR switch and magnetic-activating cell sorting (MACS), the so-called miR-switch-MACS (Tsujisaka et al., 2022). Furthermore, if one unique miRNA cannot specifically identify the cell type of interest, the more robust miRNA ON and OFF switch has been reported to enable the use of multiple miRNAs for precisely purifying such cells (Fujita et al., 2022). Since DPC-specific and HFSC-specific miRNAs have been identified (Yang et al., 2021), applying these state-of-the-art techniques for purifying hiPSC-derived trichogenic dermal cells and hiPSC-derived folliculogenic epidermal cells could possibly be performed.

In addition to the aforementioned statement, other challenges remain to be solved. If the HF components have been individually generated by hiPSCs, reconstruction of these components using an *in vitro* system such as the biomimetic developmental approach is more favorable than systems involving animals. In addition, the generation of HFs with proper surrounding tissue, blood vessels, and immune cells is desirable. Moreover, the throughput and cost of HF regeneration should match the demand, i.e., the regenerated HFs should be produced on a large scale at an affordable cost.

Although the generation of bioengineered HFs is the ultimate target for clinical application, the use of certain populations of cells may be useful and practical in some hair loss disorders. It has been reported that autologous transplantation of DSCs was useful for patients with male and female pattern hair loss (Tsuboi et al., 2020). The generation of DSCs from hiPSCs may provide an unlimited source of cells for transplantation in these patients (Ohyama, 2021). Nevertheless, bioengineered HFs are still required for some type of hair loss that involves whole HFs. Therefore, the development of a more robust platform for generating HFs through a biomimetic developmental approach, a 3D IOS devoid of animal involvement, or a skin organoid is of interest.

## 7 Conclusion

**FIGURE 6:**
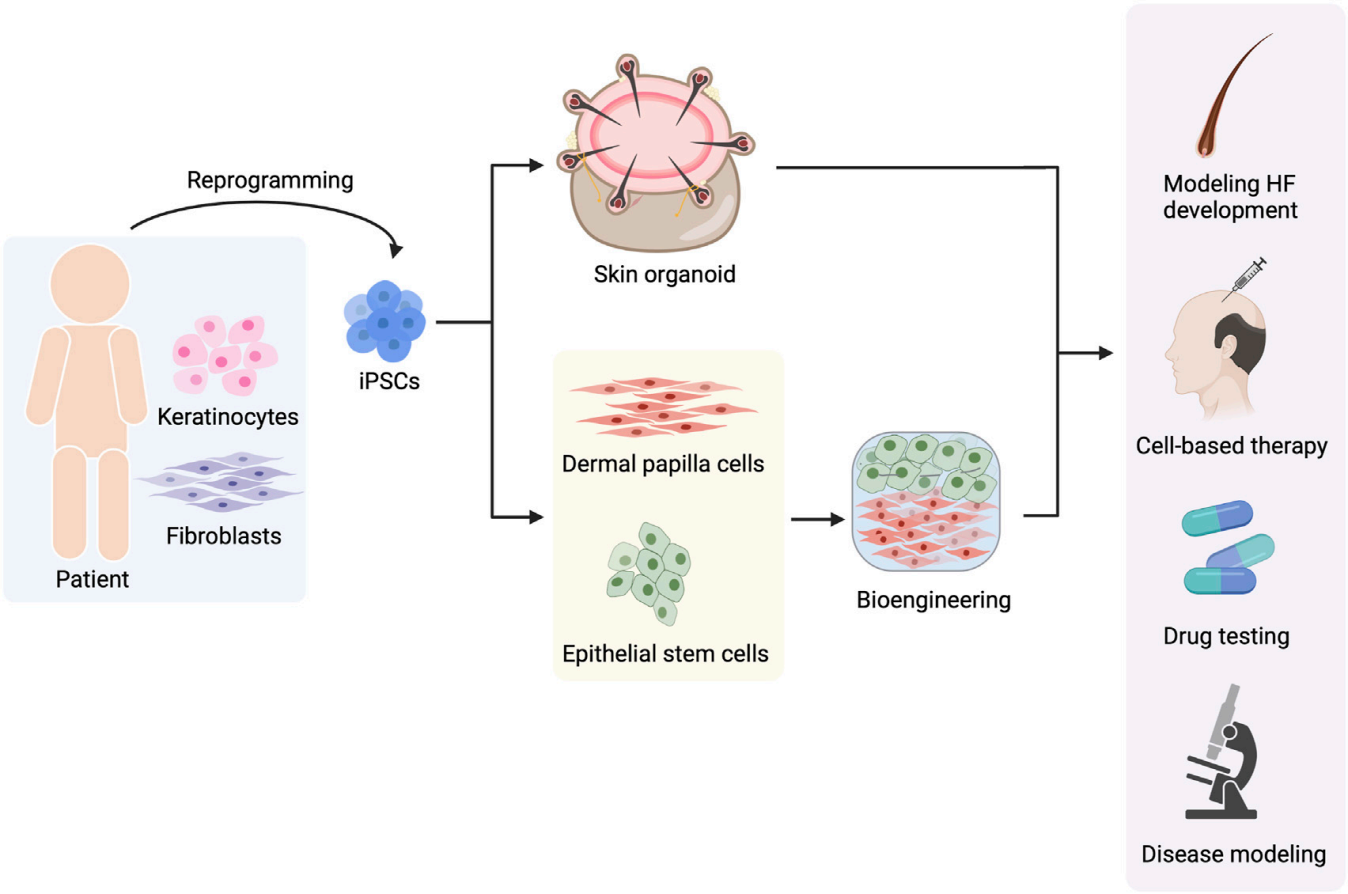
Use of hiPSCs in hair-related disorders. hiPSCs can be reprogrammed from any type of somatic cells such as keratinocytes and fibroblasts. Hair follicle (HF) regeneration can be achieved by two approaches, the generation of skin organoids and the generation of hiPSC-derived dermal papilla cells (DPCs) and hiPSC-derived epithelial stem cells (EpSCs) before gathering them to form HF using bioengineering techniques. The regenerated HFs can be useful for multiple applications including studying HF development, cell-based therapy, drug testing for hair-related diseases, and investigating the pathophysiology of hair disorders.

The recent understanding of HF biology and iPSC technology offers hope for the generation of HF components and entire HFs from hiPSCs. Several approaches for reconstructing HFs from hiPSCs have been established. However, fully functional bioengineered HFs resembling *bona fide* HFs have yet to be developed. In addition, several challenges toward the therapeutic use of regenerated HFs remain to be solved. Nevertheless, these strategies for *de novo* folliculogenesis bring us one step closer to the ultimate goal of using hiPSCs in the field of hair biology and hair disorders (Figure 6). In addition, this advancement is not only limited to cell-based therapy but also facilitates HF development research and pathogenesis studies of hair-related diseases, and enables drug discovery for hair disorders.
